# Protease Inhibitors from Plants with Antimicrobial Activity

**DOI:** 10.3390/ijms10062860

**Published:** 2009-06-23

**Authors:** Jin-Young Kim, Seong-Cheol Park, Indeok Hwang, Hyeonsook Cheong, Jae-Woon Nah, Kyung-Soo Hahm, Yoonkyung Park

**Affiliations:** 1 Research Center for Proteineous Materials, Chosun University, Gwangju 501-759, Korea; E-Mails: jyfrog@hanmail.net (J.-Y.K.); schpark9@gnu.ac.kr (S.-C.P.); kshahm@chosun.ac.kr (K.-S.H.); 2 Department of Polymer Science and Engineering, Sunchon National University, 315 Maegok, Suncheon, Korea; E-Mail: jwnah@sunchon.ac.kr (J.-W.N.); 3 Department of Cellular & Molecular Medicine, School of Medicine, Chosun University, Gwangju 501-759, Korea; 4 Department of Biotechnology and BK21 Research Team for Protein Activity Control, Chosun University, Gwangju 501-759, Korea; E-Mails: usename@hanmail.net (I.H.); hscheong@chosun.ac.kr (H.C.)

**Keywords:** plants, chromatographic columns, antimicrobial peptide, pathogenic bacterial and fungal strains, protease inhibitors, novel antimicrobial agents

## Abstract

Antimicrobial proteins (peptides) are known to play important roles in the innate host defense mechanisms of most living organisms, including plants, insects, amphibians and mammals. They are also known to possess potent antibiotic activity against bacteria, fungi, and even certain viruses. Recently, the rapid emergence of microbial pathogens that are resistant to currently available antibiotics has triggered considerable interest in the isolation and investigation of the mode of action of antimicrobial proteins (peptides). Plants produce a variety of proteins (peptides) that are involved in the defense against pathogens and invading organisms, including ribosome-inactivating proteins, lectins, protease inhibitors and antifungal peptides (proteins). Specially, the protease inhibitors can inhibit aspartic, serine and cysteine proteinases. Increased levels of trypsin and chymotrypsin inhibitors correlated with the plants resistance to the pathogen. Usually, the purification of antimicrobial proteins (peptides) with protease inhibitor activity was accomplished by salt-extraction, ultrafiltration and C_18_ reverse phase chromatography, successfully. We discuss the relation between antimicrobial and anti-protease activity in this review. Protease inhibitors from plants potently inhibited the growth of a variety of pathogenic bacterial and fungal strains and are therefore excellent candidates for use as the lead compounds for the development of novel antimicrobial agents.

## 1. Introduction

Antimicrobial peptides have been isolated from a wide variety of organisms, including animals, bacteria, insects and plants [1–6]. Innate immunity is an ancient defense strategy used by multicellular organisms to control natural flora and combat pathogens.

Plants produce compounds that act as natural defenses against pests and pathogens. Anti-microbial peptides provide the first line of defense against invading microbes in both plants and animals. Peptides ranging from 15 to 40 amino acids in length, most of which are hydrophobic and cationic, are generally involved in innate immunity. Such peptides provide protection against bacteria, fungi and viruses by acting on the cell membranes of the pathogens [7,8]. Recently, several antimicrobial plant proteins and peptides that inhibit the growth of agronomically important pathogens have been isolated from various plant sources [9]. These proteins have been divided into the following subfamilies: chitinases, β-1,3-glucanases, thaumatin-like (TL) proteins, proteinase inhibitors, endoproteinases, peroxidases, ribonuclease-like proteins, γ-thionin and plant defensins, oxalate oxidases, oxalateoxidase-like proteins and other proteins of unknown biological properties [10–16]. Protease inhibitors are ubiquitously plentiful in tubers and plant seeds [17]. Proteinase inhibitors are found in plants belonging to a variety of systematic groups, although high levels of proteinase inhibitors are often found in many plants belonging to the *Solanaceae* family [18]. Protease inhibitors in plants are usually considered to work as storage proteins (nitrogen source) and as a defense mechanism [19]. They have recently received improved interest because of their ability to potently inhibit carcinogenesis in a wide variety of *in vivo* and *in vitro* systems [20]. Several phytopathogenic fungi are known to produce extracellular proteinases [21], and recent results suggest that proteinases play an active role in the development of diseases [22]. Plants synthesize inhibitory polypeptides that can suppress the enzyme activities in response to attack by proteinases produced by phytopathogenic microorganisms [23]. This phenomenon was first recorded in tomatoes infected with *Phytophthora infestans* [24], in which increased levels of trypsin and chymotrypsin inhibitors were found to be correlated with the plants resistance to the pathogen. Later studies showed that potato tubers accumulate 20- to 24-kDa protein inhibitors of serine proteinases in response to mechanical wounding and infection with *P. infestans* [25,26]. In this review, we discuss the role of antimicrobial proteins (peptide) as protease inhibitors and their ability to overcome such resistance and emerge as a potential new class of antimicrobial agents produced from natural products [27–30].

## 2. Antimicrobial Proteins (Peptides) Produced by Various Plants

Antimicrobial peptides have been detected in a wide variety of agricultural plant species and have been implicated in the resistance of such plants to microbial infections. The localization of antimicrobial peptides in a wide range of plant tissues and their potent *in vitro* antimicrobial activity indicates that they may serve a general protective role against plant pathogens. These peptides are highly expressed both locally and systemically during pathogen attack, which supports the suggestion that they play a role in plant protection [31].

Thionins were the first plant peptides reported to have activity against plant pathogens [32]. Thionins have been shown to alter cell membrane permeability and to interact with artificial liposomes that contain phosphatidylserine. Wheat α-thionin contains 45 amino acid residues. Several families of cysteine-rich peptides have since been characterized, including defensins, lipid transfer proteins (LTPs), hevein-type peptides and knottin-type peptides [33], as well as peptide maltose binding protein (MBP)-1 from maize [34] and a group of 20-residue peptides (Ib-AMPs) isolated from the seeds of *Impatiens balsamina*.

Plant defensins, which are phylogenetically separate, but structurally similar to thionin, do not appear to cause substantial membrane permeabilization. Defensin PTH-1 from potato has 47 amino acid residues. However, there is little information available concerning possible mechanisms of action for more recently described plant peptide families, such as the so-called lipid transfer proteins (LTP), which are extracellular peptides involved in plant defense against pathogens. There is also not much information available regarding the DL1 and DL2 families (from potato tuber) of antipathogenic peptides, which may be phylogenetically related to each other and share some common features with snake-venom desintegrins [35]. From a structural point of view, LTP2 from barley is known to contain 90 amino acid residues, while no equivalent data are available for the DL1 and DL2 peptides.

Novel plant antibiotic peptides include the snakin/GASA (gibberellic acid-stimulated *Arabiopsis*) family of 12-cystein peptides, which were initially isolated from potato, but later found to be ubiquitous [36]. Novel antibiotic peptides also include shepherdins, which are linear glycine/histidine-rich peptides isolated from the roots of the shepherdins purse (*Capsella bursa-pastoris*) [37]. Finally, macrocyclic cystein-knot peptides have also been recovered from different plants belonging from the rubiaceae (coffee and other tropical plants) violacea families during screening for anti-HIV compounds [38].

Several antimicrobial peptides have been purified from potato tubers. For example, a 5-kDa Pseudothionin *Solanum tuberosum* (Pth-St1) was found to be active against bacterial and fungal pathogens of potato such as *Clavibacter michiganensis* subspecies *sepedonicus*, *Pseudomonas solanacearum* and *Fusarium solani*. Pth-St1 does not inhibit trypsin or insect α-amylase activities, and, in contrast with true thionins, it does not affect cell-free protein synthesis or β-glucuronidase activity [39]. Snakin-1 (stSN1) and Skakin-2 (stSN2) were also found to be active against the fungal pathogens, *Clavibacter michiganensis* subspecies *sepedonicus, Botrytis cinerea* at concentrations < 10 μM. Snakin-1 and Snakin-2 cause aggregation of both gram-positive and gram-negative bacteria. Snakin-1 has 63 amino acid residues (Mr 6,922), 12 of which are cysteines. In addition, Snakin-1 is unrelated to any previously isolated proteins, although it is homologous to the amino acid sequences deduced from cloned cDNAs that encode gibberellin-inducible mRNAs and has some sequence motifs that are homologous with kistrin and other hemotoxic snake venoms. For example, the corresponding StSN2 cDNA encodes a signal sequence followed by a 15-residue acidic sequence that precedes the mature StSN2 peptide, which is a basic (isoelectric point = 9.16) peptide that is 66 amino acid residues long (molecular weight of 7,025 Da) [40,41]. Finally, the potato (*Solanum tuberosum* L) tuber storage protein, patatin, was purified to homogeneity and found to have antioxidant and antiradical activity [42]. Patatin, which has a molecular mass of 45 kDa, comprises about 40% of the total soluble protein.

## 3. Three Classes of Antibiotic Peptides/Proteins from Potatoes

Based on the results of previous studies, antibiotic peptides/proteins purified from potato tubers can be divided into three classes. The first class, which includes the major proteins (peptides) in potato tubers, is composed of the globulins termed ‘tuberins’. It has recently been reported that a glycoprotein with a molecular weight of approximately 45,000 Da accounted for approximately 40% of the total soluble protein in potato; therefore, the alternate name ‘patatin’ has been widely accepted. Patatin exhibits acyl hydrolase activity as a particular phospholipase on phospholipid and lysophopholipid substrates and also acts as an esterase. In a recent study, patatin was found to have hydrolytic activity as an acidic β-1,3-glucanase. It is believed that this glucanase contributes to plant defense against fungal pathogens by digesting β-1,3-glucans in hyphal cell walls and that it is often involved in the pathogenesis-related (PR) protein response [43].

The second class of antibiotic peptides/proteins is potato defensins, which include Pthe-St1, Snakin-1 and Snakin-2. Defencins were initially isolated during extraction of the insoluble proteins produced by potato tubers and have potent antimicrobial activity. The cell wall associated peptide snakin-1 produced by the potato was initially purified as a member of what appears to be a widely distributed peptide type, the Snakin/GASA family. This peptide was found to developmentally accumulate in different tissues of potato plants and the expression of its corresponding gene was unaffected by a variety of abiotic or biotic challenges. The snakin-2 peptide, which represents a widely divergent snakin subfamily, is also active *in vitro* against bacterial and fungal plant pathogens. Furthermore, in contrast with snakin-1, expression of the gene corresponding to snakin-2 is affected by various external treatments, including pathogen infection. Although the Snakin/GASA peptides are only 38% homologous, they are basic and rich in Cys residues and have almost identical antibacterial activity spectra with aggregation of bacteria [41]. The third class of antibiotic peptides/proteins is protease inhibitors [44,45]. For example, protease inhibitors have been shown to be involved with the wound-induced defense response of plants against herbivores and pathogens [46]. They are also known to accumulate in potato leaves in response to wounding and UV irradiation [47]. Recently, protease inhibitors have received new interest due to their potent ability to prevent carcinogenesis in a wide variety of *in vivo* and *in vitro* systems [48]. Carboxypeptidase inhibitors and serine protease inhibitors from potato and other plants have also been reported to have inhibitory effects against tumor cell growth [49–51]. Moreover, by increasing the level of cholecystokinin via the inhibition of trypsin, serine protease inhibitors can be used to reduce food intake in humans [52]. Furthermore, it has been reported that carboxypeptidase inhibitor, which contains 39-amino acids and three disulfide bridges, is an antagonist of human epidermal growth factor [49]. Moreover, it was purified antimicrobial proteins (peptides) from potato, that shares homology with an acid phosphatase. Evaluation of its inhibition spectrum revealed that it has good inhibition against *Ralstonia solanacearum*, *Rhizoctonia solani* and *Alternaria solani* [53].

However, the direct effects of these antipathogenic peptides produced by potato have been poorly studied to date. In addition, there is currently no direct evidence that peptides isolated from potato exert a toxic effect on phytopathogens. Therefore, this study was conducted to evaluate low molecular-weight potato peptides with antimicrobial activity and identify the mechanism by which they exert their protective role.

## 4. Protease Inhibitors with Antimicrobial Activities from Various Plants

Protease inhibitors are ubiquitous in tubers and plant seeds [44], and are generally believed to act as storage proteins and a defense mechanism [45]. Protease inhibitors control the action of proteases that are indispensable for the growth and development of the organism. They play an important role in the protection of plant tissues from pest and pathogen attack by virtue of an antinutritional interaction. Protease inhibitors were considered to inhibit the growth of microorganism by antifeedent mechanism [54]. For example, it has been shown that protease inhibitors participate in the wound-induced defense response of plants to herbivores and pathogens [46]. Recently, protease inhibitors have received new interest due to their potent ability to prevent carcinogenesis in a wide variety of *in vivo* and *in vitro* systems [55]. In fact, serine protease inhibitors from potato and other plants have also been reported to have inhibitory effects on tumor cell growth [51,56–57]. In addition, because serine protease inhibitors can increase the level of cholecystokinin via the inhibition of trypsin, they may be useful for reducing food intake in humans [58].

For protease inhibitors to be used in humans they must be nontoxic and capable of inhibiting each of the major intestinal proteases, including pancreatic trypsin, α-chymotrypsin, and elastase. Several potentially nontoxic protease inhibitors, mostly of bacterial or plant origin, have been purified from barley seeds, cabbage leaves, and Streptomyces and are commercially available for prevention of protease-induced perianal dermatitis [59]. Because potato tuber proteins (peptides) efficiently inhibit human fecal proteases, they could also be useful for the treatment of peri-anal dermatitis [59].

### 4.1. Potide-G from Potato (Solanum tuberosum L. cv. Golden Valley)

**Figure 1.**
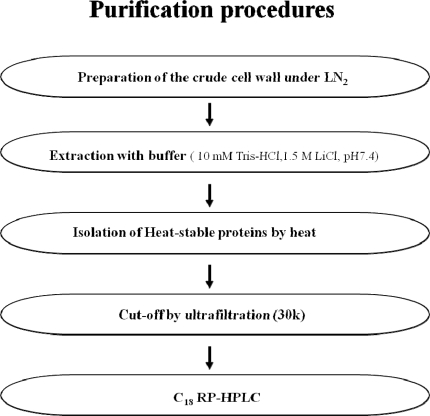
Scheme for the purification of antimicrobial peptides from potato tubers.

Potide-G is a small (5578.9 Da) antimicrobial peptide that was isolated from potato tubers (*Solanum tuberosum* L. cv. Golden Valley) (Figure 1).

**Figure 2.**
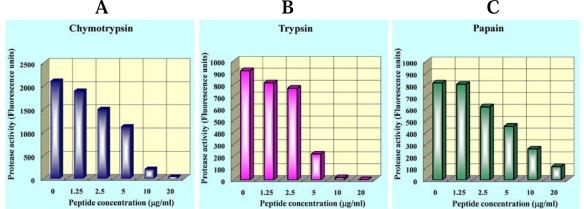
Inhibition of chymotrypsin, trypsin and papain by Potide-G. Fluorescently labeled casein was incubated at room temperature with 25 μg of the indicated enzyme for 60 min, with or without the indicated concentration of potide-G, after which the fluorescence was measured. Modified with permission from REF. 60.(2006) *Biochem Biophys Res Commun*.

**Figure 3.**
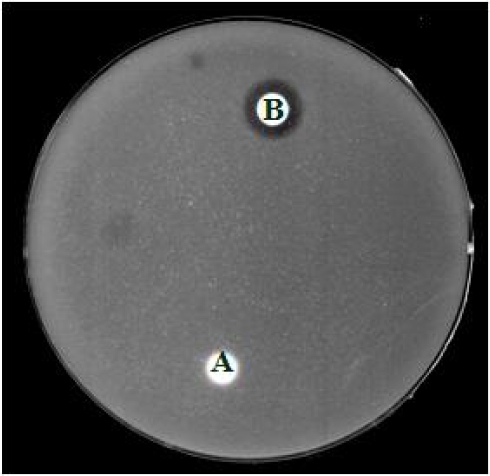
Antifungal activity of potide-G on agar containg *C. albicans.* After peptide was untreated (A) or treated (B, 5 μg) on paper discs, the plated was incubated for 24 hr at 37 °C.

This antimicrobial peptide was heat-stable and almost completely suppressed the proteolytic activity of trypsin, chymotrypsin and papain (Figure 2), with no hemolytic activity. In addition, potide-G potently inhibited the growth of a variety of bacteria (*Staphylococcus aureus*, *Listeria monocytogenes*, *Escherichia coli*, and *Clavibacter michiganense* subsp. michiganinse) and fungi (*Candida albicans* and *Rhizoctonia solani*) (Figure 3). The N-terminal sequence (residues 1 to 11) of the protein was found to be identical to that of potato proteinase inhibitor, which is a member of the Kunitz superfamily [60].

### 4.2. A napin-like Polypeptide from Dwarf Chinese White Cabbage Seeds

Napins are 1:1 disulfide-linked complexes with a smaller (ca. 4kDa) subunit and a larger (ca. 10kDa) subunit. A heterodimeric 11-kDa napin-like polypeptide has been isolated from Chinese white cabbage (Brassica chinensis cv dwarf) seeds. The N-terminal sequence of the 7-kDa subunit manifests striking similarity to the napin large chain, albumin and trypsin inhibitors. The N-terminal sequence of the 4-kDa subunit is homologous to the napin large chain and an antimicrobial peptide. Napin was found to have antibacterial activity against *Pseudomonas aeruginosia*, *Bacillus subtilis*, *Bacillus cereus*, and *Bacillus megaterium* [61].

### 4.3. Potamin-1 (PT-1), Trypsin-Chymotrypsin Protease Inhibitor Obtained from Potato

**Figure 4.**
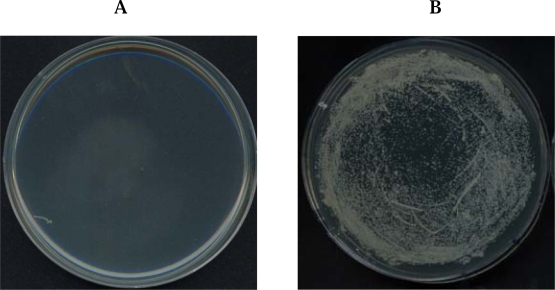
Antibacterial assay of PT-1 peptide in the absence (A) or presence (B) of DTT against *S. aureus*. After reducing the intramolecular disulfide bonds of peptide with DTT, substance was mixed with bacterial cell suspension.

A potamin-1 (PT-1) trypsin-chymotrypsin protease inhibitor was isolated from the tubers of the potato (*Solanum tuberosum* L cv. Gogu). The potamin-1 (PT-1) was thermostable and possessed antimicrobial activity, but lacked hemolytic activity. PT-1 strongly inhibited pathogenic microbial strains including *Candida albicans*, *Rhizoctonia solani*, and *Clavibacter michiganense* subsp. michiganinse. The N-terminal sequence of PT-1 was NH_2_-DICTCCAGTKGCNTTSANGAFICEG QSDPKKPKACPLNCDPHIAYA-. The sequence was 62% homologous with a serine protease inhibitor belonging to the Kunitz family, and the peptide inhibited chymotrypsin, trypsin, and papain. PT-1 was composed of polypeptide chains joined by disulfide bridge(s). Reduced PT-1 almost completely lost its activity against fungi and proteases, indicating that the disulfide bridge is essential for its protease inhibitor and antibacterial activity [62] (Figure 4). Generally, trypsin/chymotrypsin inhibitor forms to dimer by intermolecular disulfide bond. Its disulfide bond is important to maintain the antiproteolytic activity [54]. Our previous report showed that PT-1 forms a dimer by chemical crosslinking, naturally. In addition, native PT-1 showed a dimeric molecular weight on non-reducing PAGE (data not shown), meaning the formation of intermolecular disulfide bond.

### 4.4. Protein Proteinase Inhibitors in Legume Seeds

Protein proteinase inhibitors are widely distributed in plant seeds, particularly legumes. The specificity and potency of inhibition depend on defined inhibitory sites and the animal species of the target proteinase. Feeding experiments involving diets containing isolated soybean trypsin inhibitors (the Kunitz soybean trypsin inhibitor STI and the Bowman-Birk trypsinchymotrypsin inhibitor BBI) caused insignificant growth depression in rats and chicks. Furthermore, these experiments induced enlargement of the pancreas in rats, chicks and mice, but not in pigs, dogs, calves, monkeys and presumably humans. The trypsin-inhibitory site has been found to be responsible for the induction of pancreatic enlargement. Trypsin-chymotrypsin inhibitors produced by soybeans and chickpeas inhibit insect midgut proteinases, supporting the belief that proteinase inhibitors comprise a built-in defense mechanism of the seed against insects. In addition, it has been found that proteinase inhibitors such as BBI are involved in the prevention of tumorigenesis, which suggests a possible positive contribution of the inhibitors to the nutritional value of legume seeds. BBI is also an effective inhibitor of nephrotoxicity induced by the antibiotic, gentamicin. BBI does not cause side effects and does affect antimicrobial activity. Finally, it is important to note that the *in vitro* effects of proteinase inhibitors on animals should be interpreted with caution when related to humans [63].

## 5. Conclusions

Understanding the biochemical basis of the antiproteolytic and antimicrobial activities of protease inhibitors and elucidation the structure-function relationship of their activity are essential for mechanical study of their bifunctional action. Reduced protease inhibitors were found to not have antiproteolytic function or antimicrobial activity, which indicates that they are dimers in solution. These results indicate that structural changes in protease inhibitors upon manipulation of the disulfide linkage may induce a loss of both the antiproteolytic and antimicrobial activity.

Studying plant defense responses and developing newer ecofriendly strategies for protecting plants against pests and pathogens is currently one of the most dynamic areas of research in plant science. The results obtained in this study suggest that protease inhibitors are involved in the defense response of the host plant against phytopathogens. We propose that they are useful as effective antimicrobial agents and that further study is warranted. In addition, they may have the potential for use as a non-cytotoxic clinical agents.
